# Diagnostic Value of T-SPOT.TB Assay for Tuberculous Peritonitis: A Meta-Analysis

**DOI:** 10.3389/fmed.2020.585180

**Published:** 2020-12-23

**Authors:** Ying Luo, Ying Xue, Liyan Mao, Qun Lin, Guoxing Tang, Huijuan Song, Feng Wang, Ziyong Sun

**Affiliations:** ^1^Department of Laboratory Medicine, Tongji Hospital, Tongji Medical College, Huazhong University of Science and Technology, Wuhan, China; ^2^Department of Immunology, School of Basic Medicine, Tongji Medical College, Huazhong University of Science and Technology, Wuhan, China

**Keywords:** tuberculous peritonitis, T-SPOT.TB assay, diagnosis, *Mycobacterium tuberculosis*, peripheral blood, peritoneal fluid

## Abstract

**Background:** Tuberculous peritonitis (TP) is a common form of abdominal tuberculosis (TB). Diagnosing TP remains challenging in clinical practice. The aim of the present meta-analysis was to evaluate the diagnostic accuracy of peripheral blood (PB) T-SPOT and peritoneal fluid (PF) T-SPOT for diagnosing TP.

**Methods:** PubMed, EmBase, Cochrane, Scopus, Google scholar, China national knowledge internet, and Wan-Fang databases were searched for relevant articles from August 1, 2005 to July 5, 2020. Statistical analysis was performed using Stata, Revman, and Meta-Disc software. Diagnostic parameters including pooled sensitivity, specificity, positive likelihood ratio (PLR), negative likelihood ratio (NLR), and diagnostic odds ratio (DOR) were determined. Summary receiver operating characteristic curve was used to determine the area under the curve (AUC).

**Results:** Twelve studies were eligible and included in the meta-analysis. The analysis showed that the pooled sensitivity and specificity of PB T-SPOT in diagnosing TP were 0.91 (95% CI, 0.88–0.94) and 0.78 (95% CI, 0.73–0.81), respectively, while the pooled PLR, NLR, and DOR were 4.05 (95% CI, 2.73–6.01), 0.13 (95% CI, 0.07–0.23), and 37.8 (95% CI, 15.04–94.98), respectively. On the other hand, the summary estimates of sensitivity, specificity, PLR, NLR, and DOR of PF T-SPOT for TP diagnosis were 0.90 (95% CI, 0.85–0.94), 0.78 (95% CI, 0.72–0.83), 6.35 (95% CI, 2.67–15.07), 0.14 (95% CI, 0.09–0.21), and 58.22 (95% CI, 28.76–117.83), respectively. Furthermore, the AUC of PB T-SPOT and PF T-SPOT for TP diagnosis were 0.91 and 0.94, respectively.

**Conclusions:** Our results indicate that both PB T-SPOT and PF T-SPOT can be served as sensitive approaches for the diagnosis of TP. However, the unsatisfactory specificities of these two methods limit their application as rule-in tests for TP diagnosis. Furthermore, the standardization of the operating procedure of PF T-SPOT is further needed.

## Introduction

Tuberculosis (TB) remains the world's leading cause of death from a single infectious agent (1). Globally, an estimated 10.0 million people fell ill with TB, with an estimated 1.5 million TB deaths in 2018 (2). Although the most common organ affected in TB is the lung, it can also affect other organs in the body, known as extrapulmonary TB (EPTB), which represented 15% of the TB incident cases that were recognized by the World Health Organization (WHO) in 2018 (2, 3). Tuberculous peritonitis (TP) is one of the common extrapulmonary locations, accounting for around 6% of EPTB (4). Its occurrence varies according to TB prevalence, population age, and the underlying medical illness of subjects (3, 5–7). Notably, the reported mortality of this severe form can reach 30% (8–10). In countries with high TB-burden, TP appears as the one of leading cause of peritoneal fluid (PF), which justifies considering this diagnosis in all patients with peritonitis of undetermined etiology.

Early diagnosis is beneficial for anti-TB treatment, the prevention of complications, and the reduction of mortality rate. TP remains difficult to diagnose because of its non-specific clinical features and the limitations of available diagnostic tests (11, 12). The identification of *Mycobacterium tuberculosis* (MTB) in PF or tissue samples is the gold standard for TP diagnosis. However, the conventional microbiological diagnostic tests including smear microscopy, mycobacterial culture and molecular tests are seldom sensitive enough to allow for a definitive diagnosis since the disease is paucibacillary (5). More sensitive diagnosis could depend on invasive peritoneal biopsy performed by laparoscopy, which could provide a reliable means of confirming the disease histologically (13, 14). However, laparoscopic procedures are not risk-free, which may make this method not available at patients who have associated risk of complications. In addition, it may be unsuccessful in subjects with extensive adhesions (15). Besides, ultrasound and computed tomography can detect some signs in favor of TP (16). However, their infrastructure requirements and the lack of sophisticated techniques for quantification hamper the application of these techniques in the source-limited setting. Meanwhile, some routine biochemical investigations including total protein (17), lactate dehydrogenase (18), and glucose (19) in PF have extremely limited value in the diagnosis of TP due to the low sensitivities and specificities. Other indicators such as adenosine deaminase (ADA), although perform well, show poor sensitivity in cases with cirrhosis (20, 21). Interferon-gamma (IFN-γ) might present low sensitivity on diagnosing TP in patients with HIV infection (22).

As one of two commercially available interferon-gamma release assays (IGRAs), T-SPOT.TB assay (T-SPOT), which detects TB-specific cells, has been extensively applied as a diagnostic tool for pulmonary TB and EPTB in both peripheral blood (PB) and extrapulmonary samples (23–25). Due to the fact that TB-specific cells appear in PB and are recruited into the abdominal cavity during the onset of TP, PB T-SPOT, and PF T-SPOT can be used for TP diagnosis. Up to now, several studies have investigated the role of PB T-SPOT and PF T-SPOT in diagnosing TP (26, 27). However, some controversy also emerges with the introduction of this method. For example, some studies indicated that both PB T-SPOT and PF T-SPOT were not satisfactory methods for diagnosing TP (28), while some other studies indicated that PF T-SPOT presented a more prior accuracy compared with PB T-SPOT (26, 27). Hence, we performed a meta-analysis in the present study to comprehensively assess the overall accuracy of T-SPOT for TP diagnosis.

## Methods

This meta-analysis was conducted in accordance with the guidelines of the Preferred Reporting Items for Systematic Reviews and Meta-Analyses (PRISMA) statement (29). Since the study was a meta-analysis of published literatures, patient consent or approval from the institutional ethics committee was not available.

### Search Strategy

We searched for relevant individual studies published from August 1, 2005 to July 5, 2020 in PubMed database, EmBase database, Cochrane database, Scopus database, Google scholar, China national knowledge internet and Wan-Fang database, using the following search terms: (“tuberculous” or “tuberculosis” or “tubercular” or “mycobacterium” or “mycobacterial”) and (“peritonitis” or “peritoneal” or “ascites”) and (“enzyme-linked immunospot” or “ELISpot” or “T-SPOT” or “interferon-gamma release assays” or “interferon-gamma assays” or “IGRA” or “interferon release assay” or “interferon” or “interferon-gamma” or “gamma-interferon” or “T cell assays” or “T cell based assay” or “T cell response”). Relevant articles related to the keywords and the reference lists of identified publications were searched simultaneously.

### Study Selection Criteria

The inclusion criteria for relevant studies were as follows: (1) original data on the evaluation of diagnostic accuracy; (2) determinate diagnosis for TP and non-TP; and (3) sufficient data including at least sensitivity and specificity, and the number of participants. Review articles, case reports, meeting reports, and letters that did not include the original data were excluded from this study. Two reviewers (YL and YX) independently reviewed and assessed study eligibility, and disagreements were resolved by a third author (FW).

### Data Extraction and Quality Assessment

Two reviewers (YL and YX) independently extracted the following information from each study: authors, year of publication, country of origin, study design (prospective or retrospective), numbers of participants, sensitivity, specificity, and values of true-positive, false-positive, true-negative, and false-negative. The methodological quality of the studies included was assessed using the criteria of the Quality Assessment of Diagnostic Accuracy Studies-2 (QUADAS-2) (30).

### Statistical Analysis

Analysis were performed using Stata version 14.0, Revman version 5.3 and Meta-Disc version 1.4 software programs. Two-sided *P* < 0.05 was considered statistically significant. Data from individual studies were pooled using a random-effect model and used to generate values for the following measures of test accuracy: sensitivity, specificity, positive likelihood ratio (PLR); negative likelihood ratio (NLR); and diagnosis odds ratio (DOR) with corresponding 95% confidence interval (CI), a summary receiver operating characteristic (SROC) curve was made to present the individual assessment of sensitivity and specificity for each study. Sensitivity analysis was performed by focus on the risk of bias to evaluate the impact of factors with various risk on the overall results. The meta-regression analysis was used to evaluate the impact of different study designs, TB prevalence settings, and sample sizes on diagnostic accuracy of T-SPOT assay. Heterogeneity was calculated using the *I*^2^ statistic (31).

## Results

### Characteristics of the Included Studies in the Meta-Analysis

**Figure 1:**
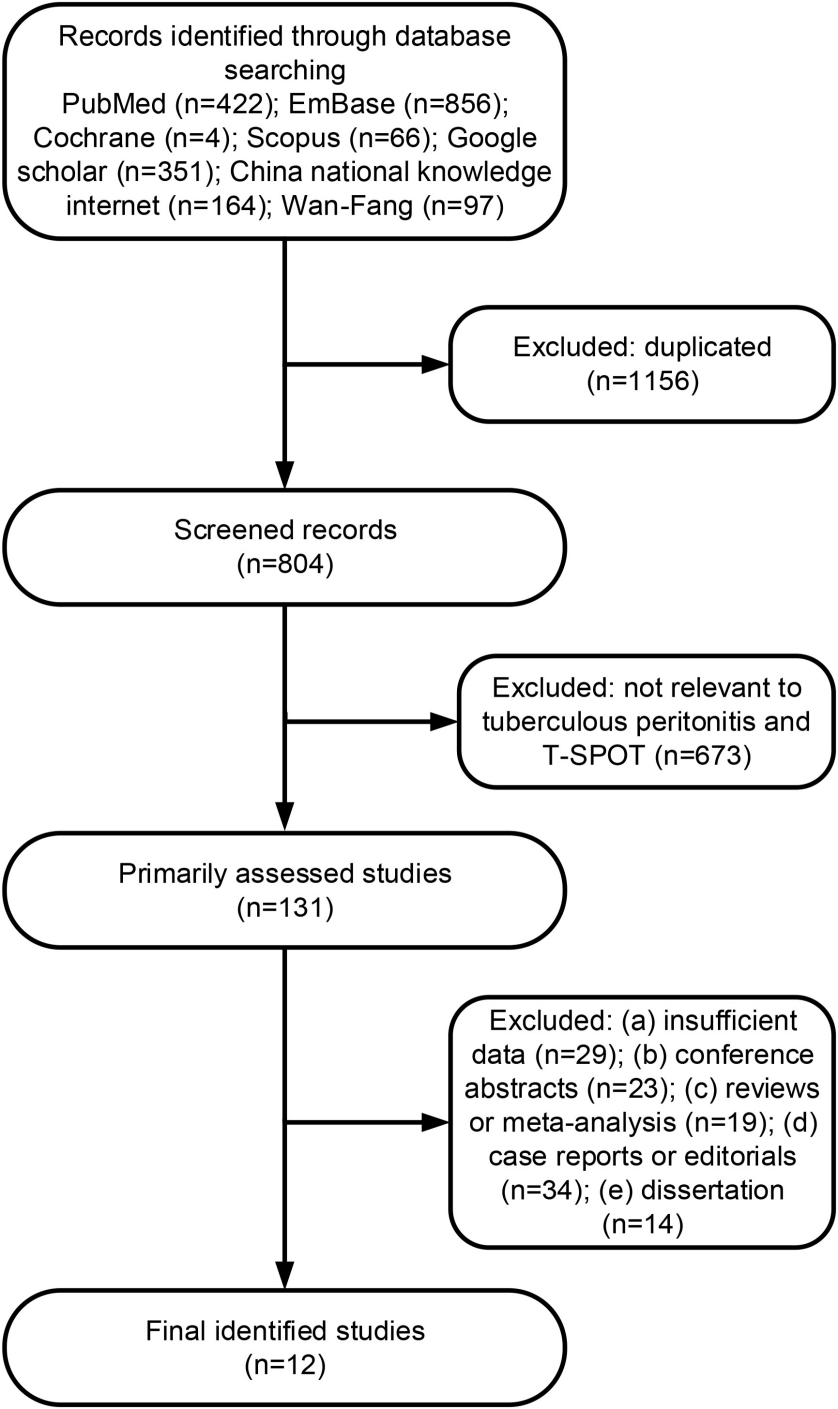
Flowchart diagram of the literature search process.

A total of 1,960 citations were initially searched (Figure 1). After independent review, we found that T-SPOT had been reported for TP diagnosis in 12 publications, which were considered eligible to be included in the meta-analysis (Table 1 and Figure 1). The PB-based T-SPOT was used in 8 studies (26–28, 33, 34, 38–40), while the PF-based T-SPOT was performed in 7 studies (26–28, 32, 35–37). Head-to-head comparisons of the diagnostic performance of PB T-SPOT against PF T-SPOT were found in 3 studies (26–28). The characteristics of these studies were summarized in Table 1. Briefly, 9 studies were performed in high TB prevalence areas (6 for PB T-SPOT and 4 for PF T-SPOT); and the other 3 studies were conducted in intermediate TB prevalence areas (2 for PB T-SPOT and 3 for PF T-SPOT). Besides, 6 studies were prospective (4 for PB T-SPOT and 4 for PF T-SPOT), while the remaining 6 were retrospective (4 for PB T-SPOT and 3 for PF T-SPOT). The number of studies performed on PB with included participants >60 was 6, while ≤60 was 2. The number of studies performed on PF with included participants >60 was 3, while ≤60 was 4. The numbers of recruited participants were 786 (352 TP and 434 non-TP) for PB T-SPOT; and 423 (171 TP and 252 non-TP) for PF T-SPOT, respectively.

**Table 1:** Summary characteristics of studies included in meta-analysis.

**Study number**	**References**	**Country**	**Continent**	TB burden*	**Study design**	**Samples**	**TP/non-TP patients recruited**	**Test results**			
								**True positive**	**False positive**	**False negative**	**True negative**
1	(32)	Korea	Asia	Intermediate	Prospective	Peritoneal fluid	6/5	5	0	1	5
2	(26)	Korea	Asia	Intermediate	Prospective	Peripheral blood	29/21	25	7	4	14
						Peritoneal fluid	12/14	11	2	1	12
3	(33)	China	Asia	High	Retrospective	Peripheral blood	37/25	36	2	1	23
4	(34)	China	Asia	High	Prospective	Peripheral blood	24/17	22	4	2	13
5	(35)	China	Asia	High	Retrospective	Peritoneal fluid	40/38	36	3	2	35
6	(36)	China	Asia	High	Retrospective	Peritoneal fluid	28/30	26	4	2	26
7	(37)	China	Asia	High	Prospective	Peritoneal fluid	18/32	17	3	4	28
8	(38)	China	Asia	High	Retrospective	Peripheral blood	55/30	54	2	1	28
9	(27)	Korea	Asia	Intermediate	Prospective	Peripheral blood	45/29	38	12	7	17
						Peritoneal fluid	45/29	39	4	6	25
10	(39)	China	Asia	High	Retrospective	Peripheral blood	68/66	62	8	6	58
11	(28)	China	Asia	High	Retrospective	Peripheral blood	21/111	16	22	5	89
						Peritoneal fluid	21/105	20	40	1	65
12	(40)	China	Asia	High	Prospective	Peripheral blood	73/135	69	40	4	95

* *Refer to Global Tuberculosis Report 2019; TB, tuberculosis; TP, tuberculous peritonitis*.

### Quality Assessment

**Figure 2:**
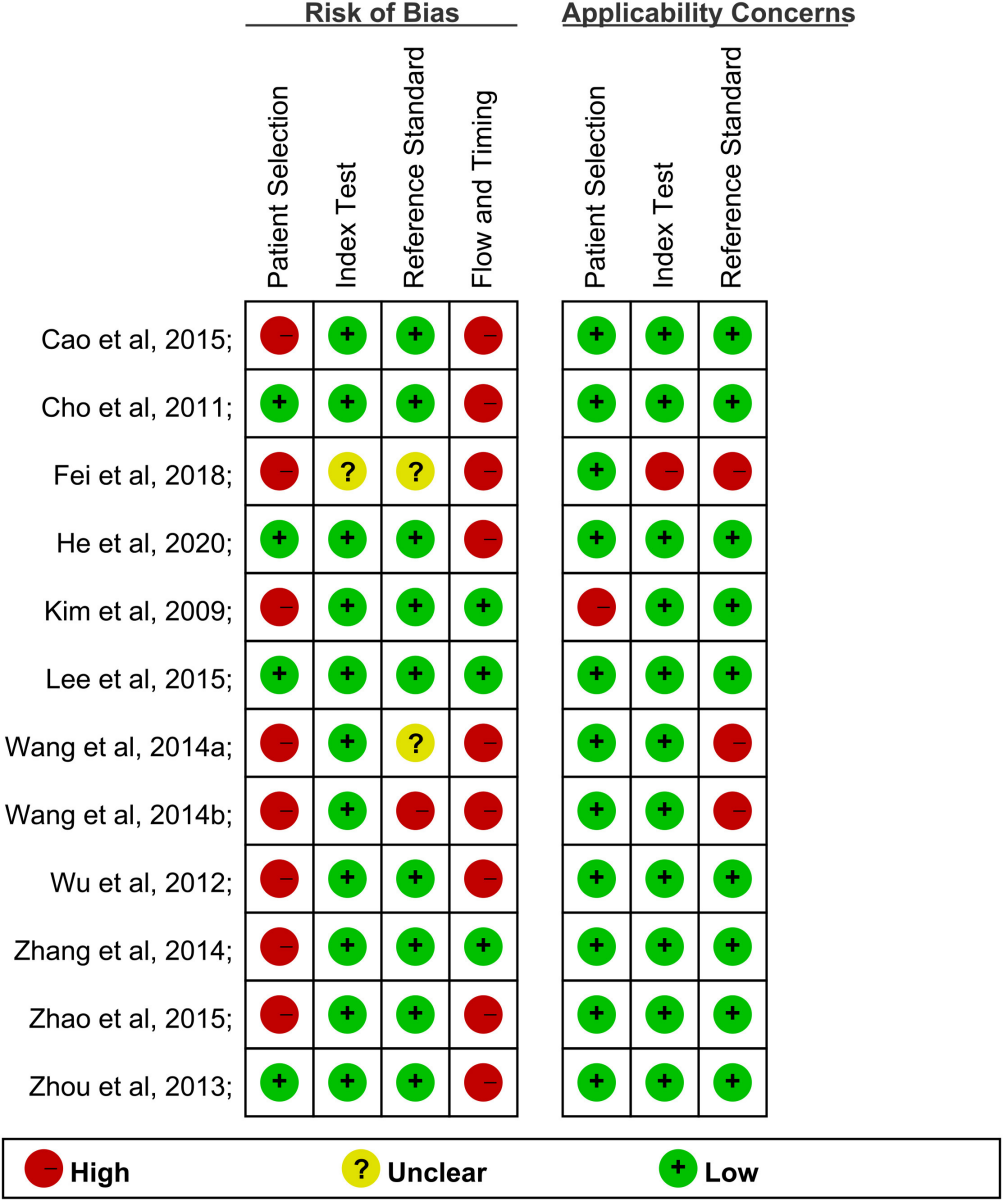
The quality of articles included.

The risk of bias and applicability concerns summary were shown in Figure 2. Only one study showed a low risk of bias (Figure 2). The risk of bias for the index test of other studies domain largely resulted from a lack of information on blinding.

### Pooled Diagnostic Accuracy of PB T-SPOT and PF T-SPOT

**Figure 3:**
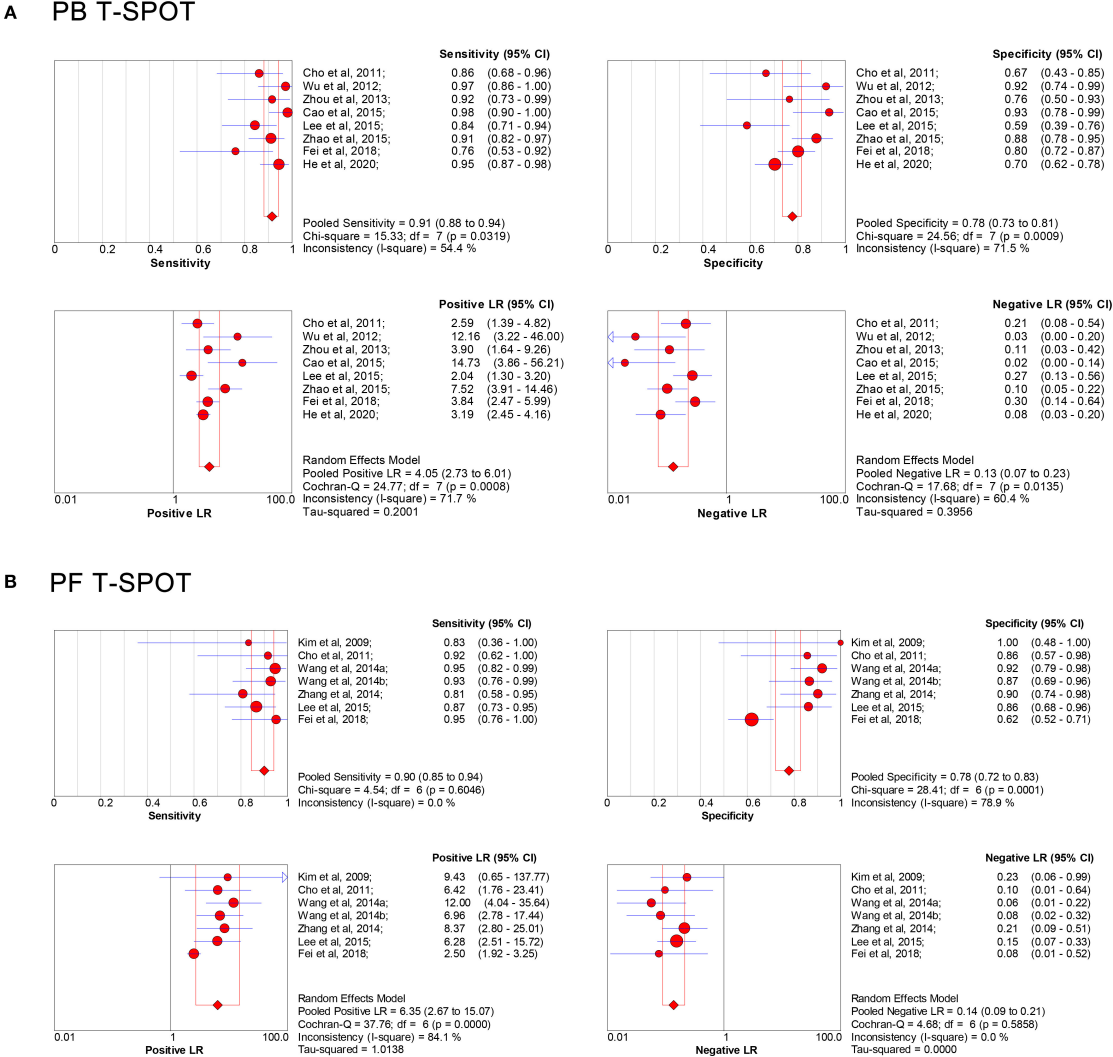
Forest plot showing estimates of sensitivity, specificity, PLR, and NLR of PB T-SPOT **(A)** and PF T-SPOT **(B)** for diagnosing TP. Point estimates of sensitivity, specificity, PLR, and NLR from each study are shown as solid circles, whose size reflects the total number of cases and controls. Error bars show 95% CI. Numbers indicate the reference numbers of studies. PLR, positive likelihood ratio; NLR, negative likelihood ratio; PB, peripheral blood; PF, peritoneal fluid; TP, tuberculous peritonitis; CI, confidence interval.

**Figure 4:**
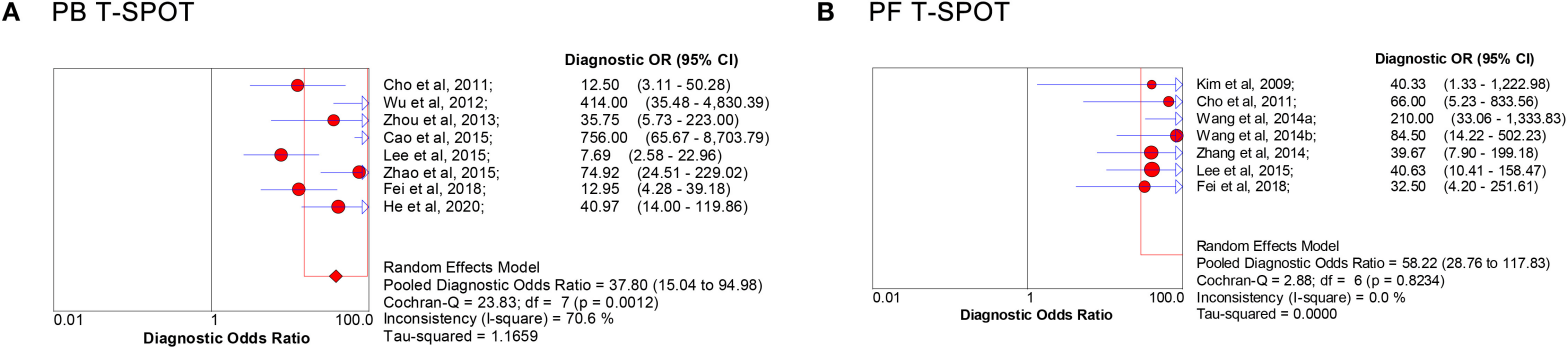
Forest plot of estimates of DOR for PB T-SPOT **(A)** and PF T-SPOT **(B)** for the diagnosis of TP. Point estimates of DOR from each study are shown as solid circles, whose size reflects the total number of cases and controls. Error bars show 95% CI. Numbers indicate the reference numbers of studies. DOR, diagnostic odds ratio; PB, peripheral blood; PF, peritoneal fluid; TP, tuberculous peritonitis.

The overall analysis showed that the pooled sensitivity, specificity, PLR, NLR, and DOR of PB T-SPOT for TP diagnosis were 91% (95% CI, 88–94%), 78% (95% CI, 73–81%), 4.05 (95% CI, 2.73–6.01), 0.13 (95% CI, 0.07–0.23), and 37.80 (95% CI, 15.04–94.98), respectively (Figures 3A, 4A). Data from the studies showed various heterogeneity for these accuracy indexes, based on *I*^2^ values of 54.4% for sensitivity, 71.5% for specificity, 71.7% for PLR, 60.4% for NLR, and 70.6% for DOR (Figures 3A, 4A).

In addition, the pooled sensitivity, specificity, PLR, NLR, and DOR of PF T-SPOT for TP diagnosis were 90% (95% CI, 85–94%), 78% (95% CI, 72–83%), 6.35 (95% CI, 2.67–15.07), 0.14 (95% CI, 0.09–0.21), and 58.22 (95% CI, 28.76–117.83), respectively (Figures 3B, 4B). Data from the studies showed various heterogeneity for these accuracy indexes, based on *I*^2^*-*values of 0.0% for sensitivity, 78.9% for specificity, 84.1% for PLR, 0.0% for NLR, and 0.0% for DOR (Figures 3B, 4B).

**Figure 5:**
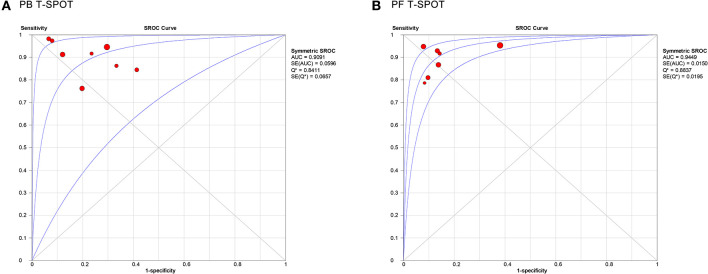
Summary receiver operating characteristic curves for PB T-SPOT **(A)** and PF T-SPOT **(B)** for diagnosing TP. PB, peripheral blood; PF, peritoneal fluid; TP, tuberculous peritonitis.

Figure 5 showed the SROC curve of T-SPOT in diagnosing TP. The area under the curve (AUC) of PB T-SPOT and PF T-SPOT for TP diagnosis were 0.9091 and 0.9449, respectively.

Meanwhile, we performed sensitivity analysis for the risk of bias on PB T-SPOT and PF T-SPOT. The results showed that the pooled sensitivity, specificity, PLR, NLR, DOR, and AUC of PB T-SPOT in studies with low risk for patient selection were 90% (95% CI, 85–94%), 69% (95% CI, 62–75%), 2.83 (95% CI, 2.23–3.58), 0.16 (95% CI, 0.08–0.30), 18.42 (95% CI, 7.63–44.44), and 0.63, respectively. The pooled sensitivity, specificity, PLR, NLR, DOR, and AUC of PB T-SPOT in studies with low risk for index test or reference standard were 92% (95% CI, 89–95%), 77% (95% CI, 72–81%), 4.23 (95% CI, 2.60–6.88), 0.11 (95% CI, 0.06–0.20), 46.58 (95% CI, 16.30–133.12), and 0.96, respectively (Supplementary Table 1).

On the other hand, the pooled sensitivity, specificity, PLR, NLR, and DOR of PF T-SPOT in studies with low risk for patient selection were 88% (95% CI, 76–95%), 86% (95% CI, 72–95%), 6.33 (95% CI, 2.99–13.37), 0.14 (95% CI, 0.07–0.29), and 45.28 (95% CI, 13.65–150.24), respectively. The pooled sensitivity, specificity, PLR, NLR, DOR, and AUC of PF T-SPOT in studies with low risk for index test were 89% (95% CI, 83–94%), 89% (95% CI, 83–94%), 7.70 (95% CI, 4.88–12.17), 0.14 (95% CI, 0.09–0.22), 62.97 (95% CI, 29.71–133.46), and 0.95, respectively. The pooled sensitivity, specificity, PLR, NLR, DOR, and AUC of PF T-SPOT in studies with low risk for reference standard were 86% (95% CI, 76–92%), 89% (95% CI, 79–95%), 7.02 (95% CI, 3.85–12.83), 0.17 (95% CI, 0.10–0.29), 42.98 (95% CI, 17.02–108.55), and 0.93, respectively. The pooled sensitivity, specificity, PLR, NLR, DOR, and AUC of PF T-SPOT in studies with low risk for flow and timing were 85% (95% CI, 74–92%), 89% (95% CI, 79–96%), 7.20 (95% CI, 3.65–14.22), 0.18 (95% CI, 0.11–0.31), 40.23 (95% CI, 14.87–108.84), and 0.93, respectively (Supplementary Table 2).

### Meta-Regression Analysis

The regression analysis was performed for T-SPOT on heterogeneous sources. It was found that the experimental design, TB burden, and the number of patients did not significantly affect the diagnostic performance of PF T-SPOT for TP (Tables 2, 3). Besides, it was observed that TB burden and the number of patients did not significantly affect the diagnostic utility of PB T-SPOT for TP (Tables 2, 3). However, the diagnostic value of PB T-SPOT for TP was significantly better in retrospective studies compared with those prospective studies (*P* = 0.04) (Tables 2, 3).

**Table 2:** Subgroup analyses for exploration of factors influencing heterogeneity in T-SPOT assay.

	**Variables**	**Category (number of studies)**	**Pooled sensitivity (95% CI)**	***I*^**2**^**	**Pooled specificity (95% CI)**	***I*^**2**^**	**Pooled diagnostic odds ratio (95% CI)**	***I*^**2**^**
PB T-SPOT	Design	Prospective (4)	0.90 (0.85–0.94)	20.9%	0.69 (0.62–0.75)	0.0%	18.42 (7.63–44.44)	45.6%
		Retrospective (4)	0.93 (0.88–0.96)	71.9%	0.85 (0.80–0.90)	44.7%	99.76 (17.85–557.54)	78.4%
	TB burden	High TB prevalence (6)	0.93 (0.90–0.96)	54.6%	0.80 (0.75–0.84)	70.7%	62.81 (22.53–175.12)	64.4%
		Intermediate TB prevalence (2)	0.85 (0.75–0.92)	0.0%	0.62 (0.47–0.75)	0.0%	9.26 (3.92–21.87)	0.0%
	Sample	>60 (6)	0.92 (0.88–0.95)	65.2%	0.78 (0.74–0.82)	78.4%	48.84 (15.06–158.33)	77.5%
		*≤* 60 (2)	0.89 (0.77–0.96)	0.0%	0.71 (0.54–0.85)	0.0%	18.37 (6.07–55.62)	0.0%
PF T-SPOT	Design	Prospective (4)	0.86 (0.76–0.92)	0.0%	0.89 (0.79–0.95)	0.0%	42.98 (17.02–108.55)	0.0%
		Retrospective (3)	0.94 (0.87–0.98)	0.0%	0.73 (0.66–0.79)	89.1%	88.41 (29.81–262.19)	0.0%
	TB burden	High TB prevalence (4)	0.92 (0.85–0.96)	11.4%	0.75 (0.69–0.81)	87.2%	68.84 (27.94–169.59)	0.0%
		Intermediate TB prevalence (3)	0.87 (0.77–0.94)	0.0%	0.88 (0.75–0.95)	0.0%	44.71 (14.42–138.61)	0.0%
	Sample	>60 (3)	0.91 (0.84–0.96)	8.9%	0.73 (0.65–0.79)	88.8%	61.87 (20.83–183.73)	18.5%
		≤ 60 (4)	0.88 (0.78–0.95)	0.0%	0.89 (0.80–0.95)	0.0%	55.67 (19.85–156.14)	0.0%

*PB, peripheral blood; PF, peritoneal fluid; TB, tuberculosis; CI, confidence interval*.

**Table 3:** Weighted meta-regression to assess the effects of various factors on diagnostic accuracy of T-SPOT assay.

	**Covariate**	**Coefficient**	**RDOR (95% CI)**	***P***
PB T-SPOT	Design			
	Prospective (4)	−4.366	0.01 (0.00–0.71)	0.04
	Retrospective (4)			
	TB burden			
	High TB prevalence (6)	0.323	1.38 (0.14–13.78)	0.69
	Intermediate TB prevalence (2)			
	Sample			
	>60 (6)	−1.305	0.27 (0.02–2.97)	0.18
	≤ 60 (2)			
PF T-SPOT	Design			
	Prospective (4)	−1.825	0.16 (0.00–96.39)	0.34
	Retrospective (3)			
	TB burden			
	High TB prevalence (4)	−0.507	0.60 (0.00–107.99)	0.71
	Intermediate TB prevalence (3)			
	Sample			
	>60 (3)	0.119	1.13 (0.03–48.43)	0.9
	≤ 60 (4)			

*PB, peripheral blood; PF, peritoneal fluid; RDOR, relative diagnostic odds ratio; TB, tuberculosis; CI, confidence interval*.

### Comparison of the Diagnostic Performance of Various Indicators for TP

In this study, we also compared the diagnostic value of T-SPOT with other tests including GeneXpert MTB/RIF, ADA, and IFN-γ from meta-analysis (41–46). Both PB T-SPOT and PF T-SPOT were more sensitive but less specific than GeneXpert MTB/RIF in diagnosing TP (Table 4). The overall accuracy of ADA and IFN-γ was superior to PB T-SPOT and PF T-SPOT (Table 4). It was observed that the sensitivities of PB T-SPOT and PF T-SPOT were comparable to both ADA and IFN-γ. However, the specificities of both two T-SPOT assays were obviously lower than those of ADA and IFN-γ (Table 4).

**Table 4:** Meta-analyses assessing the performance of GeneXpert MTB/RIF, ADA, IFN-γ, and T-SPOT for diagnosing TP.

**Biomarkers**	**References**	**TP/non-TP patients**	**Included studies**	**AUC**	**Sensitivity (95% CI)**	**Specificity (95% CI)**	**PLR (95% CI)**	**NLR (95% CI)**	**DOR (95% CI)**
GeneXpert MTB/RIF	(41)	NA	8	NA	0.30* (0.22–0.40)	1.00* (0.98–1.00)	NA	NA	41.99* (14.45–122.03)
				NA	0.64^†^ (0.49–0.76)	0.97^†^ (0.95–0.99)	NA	NA	31.43^†^ (18.88–52.33)
	(42)	115/597	16	NA	0.59 (0.45–0.74)	0.98 (0.96–0.99)	NA	NA	NA
ADA	(43)	383/1410	17	0.98	0.93 (0.90–0.95)	0.94 (0.93–0.96)	13.55 (10.22–17.97)	0.11 (0.08–0.15)	NA
	(44)	355/1219	16	0.98	0.93 (0.89–0.95)	0.96 (0.94–0.97)	15.80 (10.87–22.95)	0.09 (0.05–0.16)	249.28 (113.11–549.39)
	(45)	50/214	4	0.99	1.00 (0.93–1.00)	0.97 (0.94–0.99)	26.80 (13.30–54.00)	0.04 (0.01–0.15)	NA
IFN–γ	(46)	128/302	6	0.99	0.93 (0.87–0.97)	0.99 (0.97–1.00)	41.49 (18.80–91.55)	0.11 (0.06–0.19)	678.02 (209.91–2190.09)
PB T–SPOT	Luo et al., this study	352/434	8	0.91	0.91 (0.88–0.94)	0.78 (0.73–0.81)	4.05 (2.73–6.01)	0.13 (0.07–0.23)	37.80 (15.04–94.98)
PF T-SPOT	Luo et al., this study	171/252	7	0.94	0.90 (0.85–0.94)	0.78 (0.72–0.83)	6.35 (2.67–15.07)	0.14 (0.09–0.21)	58.22 (28.76–117.83)

* *Composite reference standard was used for TP diagnosis*;

† *culture was used as the standard for TP diagnosis; ADA, adenosine deaminase; IFN-γ, interferon-gamma; PB, peripheral blood; PF, peritoneal fluid; TP, tuberculous peritonitis; AUC, area under the curve; PLR, positive likelihood ratio; NLR, negative likelihood ratio; DOR, diagnostic odds ratio; CI, confidence interval; NA, not applicable*.

## Discussion

Date form our meta-analysis indicated that the diagnostic performance of PF T-SPOT for TP seemed comparable to PB T-SPOT, with a relatively poor specificity. The reason for the low specificity of PB T-SPOT may be that most of the studies included were performed in China; and the proportion of latent TB infection in the country is high. On the other hand, the possible reasons for decreased specificity of PF T-SPOT may be the translocation of blood TB-specific lymphocytes in latent TB infection (47, 48).

When comparing T-SPOT with other methods, it was observed that GeneXpert MTB/RIF has limited value for TP diagnosis due to the low analytical sensitivity and high cost. The meta-analysis performed by Sharma et al., showed that the overall sensitivity and specificity of GeneXpert MTB RIF in diagnosing TP were 30% and 100% when using composite reference standard for TP diagnosis, while the pooled sensitivity and specificity were 64% and 97% when using culture as the standard for TP diagnosis (41). But this method may be useful in confirming the diagnosis since it has high specificity; and it should be also considered for evaluating rifampicin resistance, which is an important drug for treating TP patients. Besides, mycobacterial culture also presented the low sensitivity of 17% with the high specificity of 100% on diagnosing TP (49). Thus, the sensitivity of both PB T-SPOT and PF T-SPOT were obviously higher than those of microbiological tests on the diagnosis of TP. Tao et al. in a meta-analysis published in 2014 found that the pooled sensitivity and specificity of ADA in diagnosing TP were 93% and 94%, respectively (43). In the study conducted by Riquelme et al., the authors found that the pooled sensitivity and specificity of ADA in diagnosing TP were 100% and 97%, respectively (45). In the meta-analysis performed by Su et al., the authors reported that the pooled sensitivity and specificity of IFN-γ on diagnosing TP were 100% and 97%, respectively (46). These results indicated that ADA and IFN-γ had good value in the diagnosis of TP. The determinations of IFN-γ and ADA are cheap and reproducible tests with the availability of results in a few hours, being especially important for routine use. However, the lack of a widely accepted cutoff value of IFN-γ caused by variable cytokine response is limiting factor for its use in routine practice. On the other hand, the fact that underlying diseases such as liver cirrhosis and immunosuppression may influence the level of ADA in patients with TP would make it difficult to determine the optimal cutoff value of ADA in different regions. In view of the low specificities and high sensitivities of PB T-SPOT and PF T-SPOT, we believe that both two methods could be combined with microbiological tests (culture or GeneXpert MTB/RIF) with high specificity to improve the diagnosis of TP. In addition, T-SPOT can also play an auxiliary role in TP diagnosis when it is difficult to obtain specimens. However, the high cost and complicated operation procedure also limits the use of T-SPOT in clinical practice.

Besides, we found that the DORs of both two assays were skyrocketing, which might be overestimated owing to the high TB prevalence in the current included studies. However, no studies performed in areas with low TB prevalence were included in the present meta-analysis. Thus, these results should be interpreted with caution. In addition, the sensitivity analysis showed that the AUC of PB T-SPOT on diagnosing TP was relatively low in studies with low risk for patient selection (0.64 vs. 0.91). However, this phenomenon should be further determined due to the low number of included studies (*n* = 4) (Table 4 and Supplementary Table 1). PB T-SPOT in studies with low risk for index test or reference standard show a higher diagnostic performance than the overall analysis, suggesting a higher diagnostic value (0.96 vs. 0.91) (Table 4 and Supplementary Table 1). On the other hand, PF T-SPOT in studies with low risk of bias presented a comparable AUC to the overall analysis (Table 4 and Supplementary Table 2). These indicated studies with good design should be further conducted to determine the performance of PB and PF T-SPOT on diagnosing TP.

In cases of active TB, abundant TB-specific cells are recruited to the morbid site (25, 32, 50, 51). T lymphocytes derived from tuberculous serous cavity effusion have been shown to proliferate and produce IFN-γ in response to TB-specific antigens *in vitro* (23, 52). Therefore, the concentration of T lymphocytes is significantly higher in PF than in PB in TP patients, resulting in that enumerating effector T-cells in PF by the enzyme linked immunospot assay would increase the sensitivity of TB diagnosis, compared with assaying PB. This evidence was supported in three head-to-head comparisons of the diagnostic performance of PB T-SPOT against PF T-SPOT (26–28). However, we did not find that the pooled sensitivity of PF T-SPOT was obviously higher than that of PB T-SPOT. This may be due to that the number of studies comparing these two methods simultaneously was a few. Most of researches separately evaluated the diagnostic performance of either PB T-SPOT or PF T-SPOT.

Another important point should be mentioned is that the results of T-SPOT performed on PF may depend on the number of PF mononuclear cells added to per well (23). As a result, it is essential to standardize the number of cells and the criteria of result interpretation to achieve consistent results to be used in clinical practice. In addition, QuantiFERON-TB Gold In-Tube, another kind of commercially available interferon-gamma release assays, were rarely reported in diagnosing TP. More data are needed on this issue in the future.

More recently, some new diagnostic approaches have been described, first, many studies reported the usefulness of interleukin-27 in the diagnosis of tuberculous pleurisy, which indicated that it may be also used for TP diagnosis (53). Second, the ratio of TB-specific antigen to phytohaemagglutinin (TBAg/PHA ratio), a new indicator introduced in T-SPOT assay, showed a helpful value in TP and other EPTB diagnosis (54). Third, GeneXpert MTB/RIF Ultra, the next generation of GeneXpert MTB/RIF, has shown an improvement compared with GeneXpert MTB/RIF (55). Therefore, this novel cartridge provides an alternative to diagnose TP in a convenient manner, and further study is urgently needed to assess its performance. Finally, the analysis of multiple cytokines of PF should be also conducted to explore the diagnostic potential. However, the current data are insufficient to decide about the exact value of these biomarkers in diagnostic algorithms. Their clinical elegance is still needed to be established in the future.

Our meta-analysis has two limitations. First, it should be noted that all studies included in the meta-analysis were from nations with intermediate or high TB-burden, which might bias the estimation of test accuracy. More prospective studies should be performed on larger cohorts in low TB incidence countries to confirm the clinical value of this assay for TP. Second, the number of studies available for inclusion was limited, with one study involving only 6 TP patients, and such small studies may be vulnerable to selection bias. Therefore, all results from the meta-analysis should be interpreted with caution and further extensive investigation is warranted to ascertain the precise diagnostic accuracy of T-SPOT in TP.

In conclusion, both PB T-SPOT and PF T-SPOT are potential complementary methods for TP diagnosis. We advocated T-SPOT coupled with other biomarkers, thereby increasing their respective value for TP diagnosis.

## Data Availability Statement

The original contributions presented in the study are included in the article/Supplementary Materials, further inquiries can be directed to the corresponding author/s.

## Author Contributions

YL, FW, and ZS designed the study, analyzed the results, and revised the manuscript. YL, YX, LM, and QL collected the data. GT and HS helped to design the data abstraction form. All authors reviewed the final manuscript.

## Conflict of Interest

The authors declare that the research was conducted in the absence of any commercial or financial relationships that could be construed as a potential conflict of interest.

## Abbreviations

ADA: adenosine deaminase
EPTB: extrapulmonary tuberculosis
Xpert: GeneXpert MTB/RIF
IFN-γ: interferon-gamma
IGRAs: interferon-gamma release assays
MTB: *Mycobacterium tuberculosis*
PB: peripheral blood
PF: peritoneal fluid
TB: tuberculosis
TP: tuberculous peritonitis
TBAg/PHA ratio: tuberculosis-specific antigen to phytohaemagglutinin
T-SPOT: T-SPOT.TB assay
WHO: World Health Organization.
