# Supplementary material for: Diagnostic Value of T-SPOT.TB Assay for Tuberculous Peritonitis: A Meta-Analysis
Source: Front Med (Lausanne). 2020 Dec 23;7:585180. doi: 10.3389/fmed.2020.585180 (PMC7785855; doi:10.3389/fmed.2020.585180)
Supplement: Supplementary file 1 [file Table_1.DOCX]

| **Supplementary Table 1. Results of sensitivity analysis for PB T-SPOT on diagnosing TP.** | | | | |
| --- | --- | --- | --- | --- |
|  | Low risk for patient selection | Low risk for index test | Low risk for reference standard | Low risk for flow and timing |
| Number of studies | 4 | 7 | 7 | 1 |
| AUC | 0.63 | 0.96 | 0.96 | NA |
| Pooled sensitivity (95% CI) | 0.90 (0.85-0.94) | 0.92 (0.89-0.95) | 0.92 (0.89-0.95) | NA |
| *I*^2^ | 20.9% | 42.9% | 42.9% | NA |
| Pooled specificity (95% CI) | 0.69 (0.62-0.75) | 0.77 (0.72-0.81) | 0.77 (0.72-0.81) | NA |
| *I*^2^ | 0.0% | 75.0% | 75.0% | NA |
| Pooled PLR (95% CI) | 2.83 (2.23-3.58) | 4.23 (2.60-6.88) | 4.23 (2.60-6.88) | NA |
| *I*^2^ | 11.5% | 76.0% | 76.0% | NA |
| Pooled NLR (95% CI) | 0.16 (0.08-0.30) | 0.11 (0.06-0.20) | 0.11 (0.06-0.20) | NA |
| *I*^2^ | 44.9% | 56.8% | 56.8% | NA |
| Pooled DOR (95% CI) | 18.42 (7.63-44.44) | 46.58 (16.30-133.12) | 46.58 (16.30-133.12) | NA |
| *I*^2^ | 45.6% | 71.9% | 71.9% | NA |
| PB, peripheral blood; TP, tuberculous peritonitis; AUC, area under the curve; PLR, positive likelihood ratio; NLR, negative likelihood ratio; DOR, diagnostic odds ratio; NA, not applicable. | | | | |
