# Supplementary material for: Diagnostic Value of T-SPOT.TB Assay for Tuberculous Peritonitis: A Meta-Analysis
Source: Front Med (Lausanne). 2020 Dec 23;7:585180. doi: 10.3389/fmed.2020.585180 (PMC7785855; doi:10.3389/fmed.2020.585180)
Supplement: Supplementary file 2 [file Table_2.DOCX]

| **Supplementary Table 2. Results of sensitivity analysis for PF T-SPOT on diagnosing TP.** | | | | |
| --- | --- | --- | --- | --- |
|  | Low risk for patient selection | Low risk for index test | Low risk for reference standard | Low risk for flow and timing |
| Number of studies | 2 | 6 | 4 | 3 |
| AUC | NA | 0.95 | 0.93 | 0.93 |
| Pooled sensitivity (95% CI) | 0.88 (0.76-0.95) | 0.89 (0.83-0.94) | 0.86 (0.76-0.92) | 0.85 (0.74-0.92) |
| *I*^2^ | 0.0% | 0.0% | 0.0% | 0.0% |
| Pooled specificity (95% CI) | 0.86 (0.72-0.95) | 0.89 (0.83-0.94) | 0.89 (0.79-0.95) | 0.89 (0.79-0.96) |
| *I*^2^ | 0.0% | 0.0% | 0.0% | 0.0% |
| Pooled PLR (95% CI) | 6.33 (2.99-13.37) | 7.70 (4.88-12.17) | 7.02 (3.85-12.83) | 7.20 (3.65-14.22) |
| *I*^2^ | 0.0% | 0.0% | 0.0% | 0.0% |
| Pooled NLR (95% CI) | 0.14 (0.07-0.29) | 0.14 (0.09-0.22) | 0.17 (0.10-0.29) | 0.18 (0.11-0.31) |
| *I*^2^ | 0.0% | 0.0% | 0.0% | 0.0% |
| Pooled DOR (95% CI) | 45.28 (13.65-150.24) | 62.97 (29.71-133.46) | 42.98 (17.02-108.55) | 40.23 (14.87-108.84) |
| *I*^2^ | 0.0% | 0.0% | 0.0% | 0.0% |
| PF, peritoneal fluid; TP, tuberculous peritonitis; AUC, area under the curve; PLR, positive likelihood ratio; NLR, negative likelihood ratio; DOR, diagnostic odds ratio; NA, not applicable. | | | | |
